# A novel IRS-1-associated protein, DGKζ regulates GLUT4 translocation in 3T3-L1 adipocytes

**DOI:** 10.1038/srep35438

**Published:** 2016-10-14

**Authors:** TingYu Liu, BuChin Yu, Mamoru Kakino, Hitoshi Fujimoto, Yasutoshi Ando, Fumihiko Hakuno, Shin-Ichiro Takahashi

**Affiliations:** 1 Departments of Animal Sciences and Applied Biological Chemistry, Graduate School of Agriculture and Life Sciences, The University of Tokyo, 1-1-1 Yayoi, Bunkyo-ku, Tokyo, Japan

## Abstract

Insulin receptor substrates (IRSs) are major targets of insulin receptor tyrosine kinases. Here we identified diacylglycerol kinase zeta (DGKζ) as an IRS-1-associated protein, and examined roles of DGKζ in glucose transporter 4 (GLUT4) translocation to the plasma membrane. When DGKζ was knocked-down in 3T3-L1 adipocytes, insulin-induced GLUT4 translocation was inhibited without affecting other mediators of insulin-dependent signaling. Similarly, knockdown of phosphatidylinositol 4-phosphate 5-kinase 1α (PIP5K1α), which had been reported to interact with DGKζ, also inhibited insulin-induced GLUT4 translocation. Moreover, DGKζ interacted with IRS-1 without insulin stimulation, but insulin stimulation decreased this interaction. Over-expression of sDGKζ (short-form DGKζ), which competed out DGKζ from IRS-1, enhanced GLUT4 translocation without insulin stimulation. Taking these results together with the data showing that cellular PIP5K activity was correlated with GLUT4 translocation ability, we concluded that IRS-1-associated DGKζ prevents GLUT4 translocation in the absence of insulin and that the DGKζ dissociated from IRS-1 by insulin stimulation enhances GLUT4 translocation through PIP5K1α activity.

It is well known that insulin is a major anabolic hormone, which maintains glucose homeostasis by stimulating glucose uptake and utilization in muscle and adipose tissues and suppressing glucose production in liver. Glucose uptake in muscle and adipose tissues is induced by translocation of glucose transporter 4 (GLUT4) from multiple intracellular compartments to the plasma membrane (PM). In general, insulin, binding to its specific receptor on the plasma membrane, induces activation of intrinsic tyrosine kinase activity^1^,^2^. Activated receptor phosphorylates several intracellular substrates including insulin receptor substrates (IRSs). Tyrosine phosphorylation of IRSs leads to their binding to several intermediate signaling molecules containing SH2 domains including a p85 phosphatidylinositol (PI) 3-kinase regulatory subunit and Grb2. These bindings allow activation of the distinct signaling pathways, PI 3-kinase cascade and Ras-MAPK cascade. In particular, activation of PI 3-kinase leads to activation of Ser/Thr kinase, Akt. Activated Akt kinase phosphorylates some substrates including AS160 which regulated the GLUT4 translocation from intracellular vesicles to the PM and subsequent glucose uptake^3^,^4^,^5^,^6^,^7^,^8^. Thus IRS-1 is an important mediator of insulin signal activation required for GLUT4 translocation and glucose uptake.

Recently, however, we found that IRS-1 formed high-molecular mass complexes in 3T3-L1 adipocytes not through recognition of tyrosine phosphorylation^9^. Furthermore, we and other groups have shown that some IRS-associated proteins (IRSAPs), Nexilin, PHIP, 53BP2S, HSP90, Nedd4 or GKAP42 modulated insulin/insulin-like growth factor (IGF)-dependent tyrosine phosphorylation of IRS-1 or IRS-2 in insulin/IGF targeted cells^10^,^11^,^12^,^13^,^14^,^15^. Further identification of other IRS-associated proteins and elucidation of their roles are essential prerequisites for understanding alternative mechanisms of modulation of insulin-mediated signals and bioactivities.

In this study, we identified diacylglycerol kinase (DGK) ζ as a novel IRS-1-associated protein. DGKζ belongs to type IV among five DGK family groups^16^,^17^. Each type of DGK has a unique character in terms of regulatory mechanism, binding protein, and subcellular localization. DGK had been reported to catalyze phosphorylation of diacylglycerol (DAG) to generate phosphatidic acid (PA) and to regulate various cellular responses by changing the balance between DAG and PA levels^18^,^19^,^20^. DGKζ contains two DAG binding domains (C1 domain), the myristoylated alanine-rich C kinase substrate (MARCKs) domain, catalytic domain and ankyrin repeat domain^21^. Recently it was reported that DGKζ had roles in regulation of the transcription factors, p53 and NF-κB^22^,^23^,^24^. DGKζ had been reported to interact and form complexes with certain enzymes, which are activated by DAG (Ras-GRP, cPKC) or PA (PIP5K1α), suggesting that DGKζ complex formation might regulate the direction or the balance of signals activated by DAG or PA^25^,^26^,^27^,^28^. In this study, the role of PIP5K1α was also investigated.

Our present data indicated that both DGKζ and PIP5K1α regulated GLUT4 translocation to the PM by a novel mechanism. Interestingly, under basal conditions, DGKζ and IRS-1 interacted with each other, and IRS-1-associated DGKζ inhibited GLUT4 translocation. Once insulin signaling was activated, DGKζ was dissociated from IRS-1 and this DGKζ enhanced GLUT4 translocation through PIP5K1α activity.

## Results

### We identified diacylglycerol kinase zeta (DGKζ) as a novel IRS-1-associated protein

**Figure 1:**
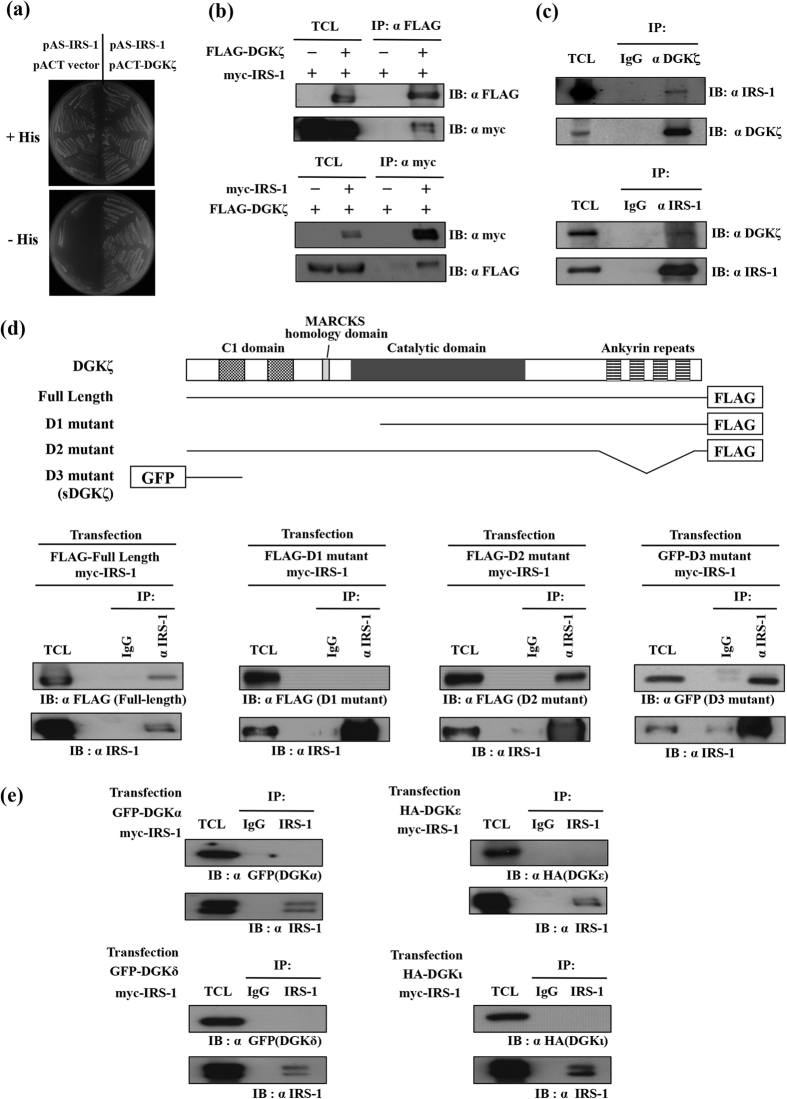
Interaction of DGKζ with IRS-1. (**a**) pAS-IRS-1 which expresses full length of IRS-1 fused with Gal4-DNA binding domain was used as bait. pACT-DGKζ which expresses N-terminal fragment of DGKζ fused with Gal4-activation domain was used as prey. Yeast strain, AH109 was used for the two-hybrid assay. Yeast transformants were grown on the medium lacking leucine and tryptophan (SD + His) or the medium lacking leucine, tryptophan and histidine (SD-His). (**b**) HEK293T cells were transfected with pFLAG-DGKζ (expressing FLAG-tagged DGKζ) and pmyc-IRS-1 (expressing myc-tagged IRS-1). Cells were serum-starved for 4 h and cell lysates were prepared (TCL). Cell lysates were immunoprecipitated by FLAG-agarose beads or anti-myc antibody. TCL and immunoprecipitates^14^ were subjected to immunoblotting analysis with indicated antibodies. (**c**) Fully differentiated 3T3-L1 adipocytes were serum-starved for 4 h and TCL were prepared. Cell lysates were immunoprecipitated by anti-DGKζ antibody, anti-IRS-1 antibody or control IgG respectively. TCL or IP were subjected to immunoblotting analysis using indicated antibody. (**d**) A schematic structure of DGKζ protein is shown. Two C1 domains are shown in dotted boxes. Myristoylated alanine-rich C kinase substrate (MARCKs) domains indicated by the light grey box. The dark grey box indicates the catalytic domain and ankyrin repeat domain is shown in striped boxes. Full-length DGKζ and three deletion constructs of DGKζ (D1, D2 and D3) were tagged with FLAG or GFP. Each mutant and myc-IRS1 was co-expressed in HEK293T cells. Cell lysates were immunoprecipitated with anti-IRS-1 antibody or control IgG. IP or TCL were subjected to immunoblotting analysis using anti-FLAG or GFP antibody. (**e**) GFP-DGKα, GFP-DGKδ, HA-DGKε or HA-DGKι was co-expressed with myc-IRS-1 in HEK293T cells. Cell lysates were immunoprecipitated with anti-IRS-1 antibody and the interaction was detected with indicated antibody.

To identify proteins, which interact with IRS-1 and play some roles in GLUT4 translocation, we searched for IRS-1-associated proteins from a cDNA library of 3T3-L1 adipocytes by yeast two-hybrid screening utilizing full-length IRS-1 as a bait. Through this screening we identified 14-3-3 family proteins, μ chain of AP-1 complex protein and 53BP2S/ASPP2, which had been already reported^11^,^12^,^29^. In addition to these proteins, we identified diacylglycerol kinase zeta (DGKζ) as a novel IRS-1-associated protein. The interaction of DGKζ with IRS-1 was confirmed by yeast two-hybrid assay (Fig. 1a). The interaction between DGKζ and IRS-1 was detected in co-immunoprecipitation assays (Fig. 1b). Next, to confirm the interaction of endogenous IRS-1 and DGKζ, cell lysates of fully differentiated 3T3-L1 adipocytes were immunoprecipitated with anti-DGKζ or anti-IRS-1 antibody followed by immunoblotting analysis using anti-IRS-1 or anti-DGKζ antibody. As shown in Fig. 1c, the interaction between endogenously expressed IRS-1 and DGKζ was detected in 3T3-L1 adipocytes. Next, to define the regions of DGKζ required for interaction with IRS-1, we made deletion constructs of DGKζ and the interaction was examined by co-immunoprecipitation assay. Mutants which contained the C1 domain (full length, D2 and D3 mutant) could interact with IRS-1. But a mutant that lacks the C1 domain (D1 mutant) could not interact with IRS-1 (Fig. 1d). These data indicated that the N-terminal region containing the C1 domain is responsible for the interaction with IRS-1. Based on structural motifs, all DGK family proteins have C1 domains. Then we examined whether the other DGK family proteins could interact with IRS-1. Surprisingly, DGKα, DGKβ, DGKγ, DGKδ, DGKε, DGKη and DGKι did not interact with IRS-1 even though these isoforms have the C1 domain. Only DGKζ among various DGKs could interact with IRS-1 (Fig. 1e and Supplemental Fig. S1).

### Knockdown or overexpression of DGKζ modulates GLUT4 translocation

**Figure 2:**
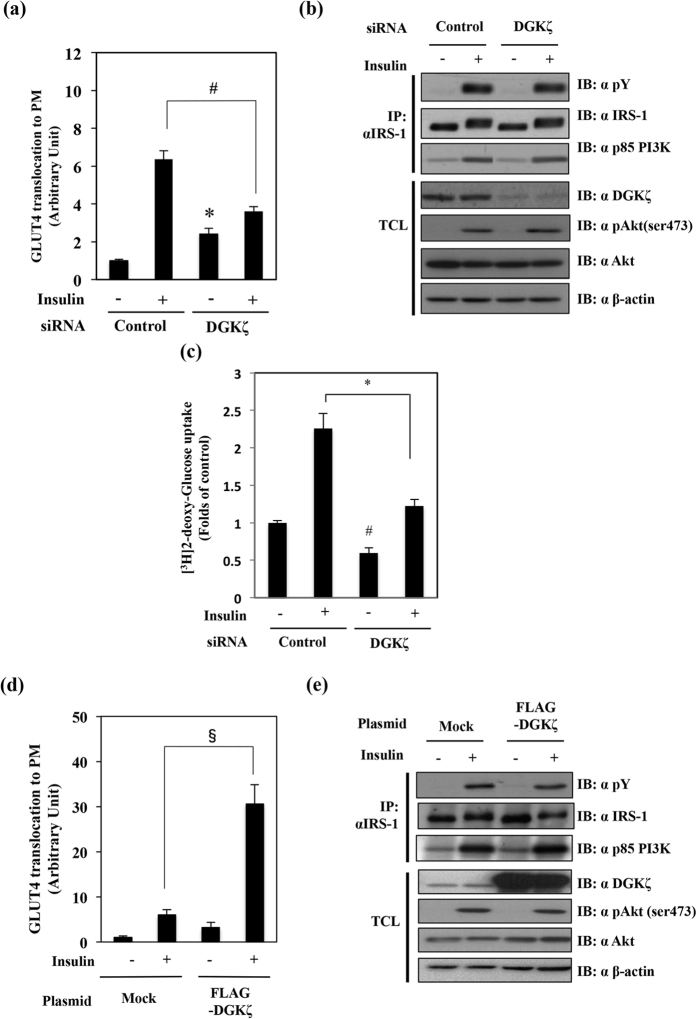
Effects of knockdown or overexpression of DGKζ on the GLUT4 translocation, glucose uptake and insulin signaling. (**a**) Fully differentiated 3T3-L1 adipocytes were electroporated with non-relevant control siRNA (control) or DGKζ siRNA together with the pGLUT4-myc-GFP plasmid. Beginning 24 h after the electroporation the cells were serum-starved for 4 h and treated with or without insulin (100 nM) for 20 min. Cells were fixed without permeabilization and stained with anti-myc antibody as described in the Materials and Methods. The ratio of (exofacial-exposed Myc epitope fluorescence/total GFP fluorescence) was quantified. The results are presented as the means ± S.E.M. of twenty cells. (**b**) Cell lysates were immunoprecipitated with the indicated antibodies, and IP or TCL were immunoblotted with the indicated antibodies. (**c**) 3T3-L1 adipocytes were electroporated with control siRNA or DGKζ siRNA were starved for 16–24 h, and followed by stimulation of 100 nM insulin for 20 min. Then 0.1 mM 2-deoxy-D-glucose containing 10 μCi/ml 2-deoxy-D-[2,6-^3^H] glucose was added, and cells were incubated for 4 min at 37 °C. These cells were harvested for glucose uptake as described in the methods. (**d**) 3T3-L1 adipocytes were electroporated with mock vector or pFLAG-DGKζ together with pGLUT4-myc-GFP plasmids. Cells were serum-starved for 4 h, followed by stimulation with insulin. GLUT4 translocation was then measured. (**e**) CHO cells were transfected with mock vector or pFLAG-DGKζ plasmid. TCL from transfectants were immunoprecipitated with the anti-IRS-1 antibody. IP or TCL were immunoblotted with the indicated antibodies. These are representative data from experiments independently performed at least three times. *p < 0.05 as compared with control group. ^#^*p* < 0.05 as compared with control plus insulin treatment group. ^§^*p* < 0.05 as compared with the mock plus insulin treatment group.

Because insulin-induced GLUT4 translocation is an important event, we next examined the effects of knockdown of DGKζ on insulin-dependent GLUT4 translocation and signaling activation in 3T3-L1 adipocytes. As shown in Fig. 2a, insulin stimulation resulted in an approximately 6-fold increase in the control cells. However, DGKζ knockdown significantly suppressed insulin-induced GLUT4 translocation to the PM. Interestingly, under basal conditions (without insulin stimulation), DGKζ knockdown enhanced the level of GLUT4 translocation, suggesting that DGKζ plays some roles in GLUT4 translocation even under basal conditions. Next, the effects of DGKζ knockdown on the insulin signaling pathways were investigated. 3T3-L1 adipocytes were electroporated with siRNA against DGKζ, resulting in a marked reduction in the DGKζ protein level without any significant effect on the levels of other proteins (Fig. 2b). The total cell lysates from DGKζ knockdown 3T3-L1 adipocytes were immunoprecipitated with anti-IRS-1 antibody, and assessed for insulin-induced IRS-1 tyrosine phosphorylation or binding of the p85 PI 3-kinase regulatory subunit to IRS-1. As shown in Fig. 2b, we found that DGKζ knockdown did not affect insulin-induced IRS-1 tyrosine phosphorylation nor binding of the p85 PI 3-kinase regulatory subunit to IRS-1. Consistent with these effects of DGKζ knockdown, the levels of insulin-induced Akt phosphorylation were also not affected (Fig. 2b). As shown in Fig. 2c, glucose uptake was significantly suppressed in DGKζ knockdown adipocytes under both basal and insulin-stimulated conditions. We then examined the effects of DGKζ overexpression on the insulin-induced GLUT4 translocation. As shown in Fig. 2d, DGKζ overexpression significantly enhanced the insulin-induced GLUT4 translocation in 3T3-L1 adipocytes. Furthermore, we measured the insulin signal activation in DGKζ-overexpressed cells. Because of low transfection efficiency by electroporation into 3T3-L1 cells, we used CHO cells which have similar signal transduction to 3T3-L1, and are abundantly used for transfection^30^,^31^. The insulin-induced IRS-1 tyrosine phosphorylation and association with p85 PI 3-kinase regulatory subunit and Akt phosphorylation were not affected in the DGKζ-overexpressing cells (Fig. 2e).

### Knockdown or overexpression of PIP5K1α modulates the insulin-induced GLUT4 translocation

**Figure 3:**
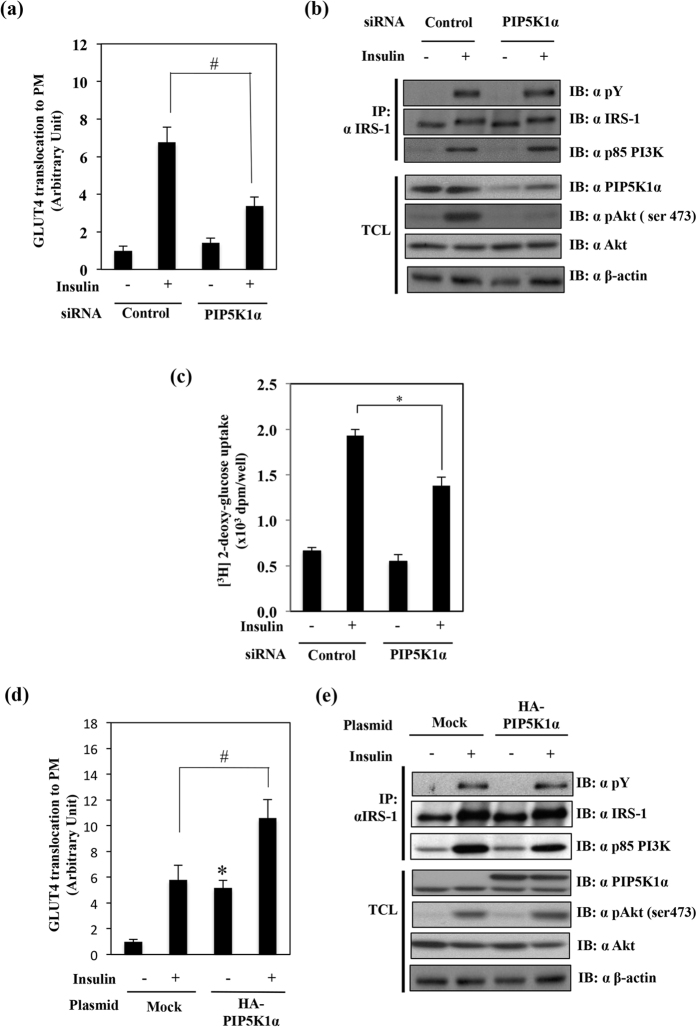
Effects of knockdown or overexpression of PIP5K1α on GLUT4 translocation, glucose uptake and insulin signaling. (**a**) 3T3-L1 adipocytes were electroporated with non-relevant siRNA (control) or PIP5K1α siRNA along with the pGLUT4-myc-GFP plasmid. GLUT4 translocation was measured as described in Fig. 2a. The results are presented as the means ± S.E.M. of twenty cells. (**b**) Fully differentiated 3T3-L1 adipocytes were electroporated with control siRNA or PIP5K1α siRNA. Activation of the canonical insulin signal pathway was assessed as described in Fig. 2b. (**c**) 3T3-L1 adipocytes were electroporated with control siRNA or PIP5K1α siRNA were starved for 16–24 h, and followed by stimulation with insulin. These cells were harvested for glucose uptake as described in Fig. 2c. (**d**) 3T3-L1 adipocytes were electroporated with mock or pHA-PIP5K1α together with pGLUT4-myc-GFP plasmids followed by insulin treatment. GLUT4 translocation was measured as described in Fig. 2a. (**e**) CHO cells were transfected with mock or pHA-PIP5K1α plasmid. Activation of insulin signaling was examined as described in Fig. 2e. These are representative data from experiments independently performed at least three times. ^#^*p* < 0.05 as compared with control plus insulin treatment group. *p < 0.05 as compared with control group.

PIP5K1α had been reported to interact with DGKζ^27^. Moreover, PIP5K1α was activated by PA, which is produced by DGKζ activity^27^. These results led us to assess the roles of PIP5K1α in insulin-induced GLUT4 translocation to the PM. We knocked down PIP5K1α in 3T3-L1 adipocytes and measured the GLUT4 translocation and signal activation. As shown in Fig. 3a, PIP5K1α knockdown significantly suppressed insulin-induced GLUT4 translocation to PM. Under this condition, insulin-stimulated IRS-1 tyrosine phosphorylation and association with the p85 PI 3-kinase regulatory subunit were not affected. However, the Akt phosphorylation was significantly suppressed (Fig. 3b). As shown in Fig. 3c, insulin-induced glucose uptake was also significantly suppressed in PIP5K1α knockdown adipocytes. We then examined the effects of PIP5K1α overexpression on GLUT4 translocation using 3T3-L1 adipocytes transfected with a HA-PIP5K1α expressing plasmid together with pGLUT4-myc-GFP plasmid. As shown in Fig. 3d, the PIP5K1α overexpression enhanced GLUT4 translocation both under basal and insulin stimulated conditions. We then transfected CHO cells with HA-PIP5K1α expressing plasmids and measured the insulin signal activation. The insulin-dependent IRS-1 tyrosine phosphorylation and association with p85 PI3K regulatory subunit and Akt phosphorylation were not changed in PIP5K1α overexpressing cells (Fig. 3e).

### Insulin stimulation dissociated DGKζ and PIP5K1α from IRS-1

**Figure 4:**
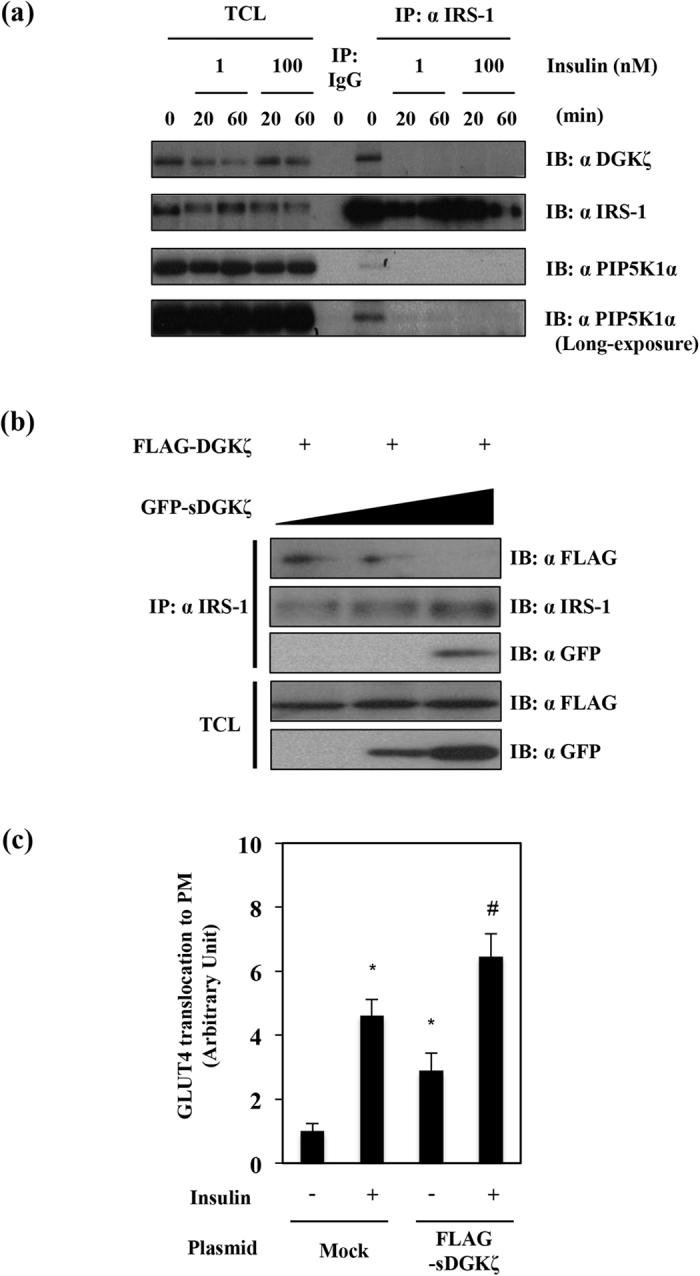
Effects of insulin treatment or sDGKζ overexpression on interaction between DGKζ and IRS-1 and GLUT4 translocation. (**a**) CHO cells were serum-starved for 18 h followed by treatment with insulin (1 nM or 100 nM) for 20 and 60 min, and then TCL were immunoprecipitated with anti-IRS-1 antibody or control IgG. TCL and IP were subjected to immunoblotting analysis with indicated antibodies. (**b**) HEK293T cells were transfected with pFLAG-DGKζ plasmid and different amounts of pGFP-sDGKζ plasmid. Cell lysates were prepared from each cell treatment and were immunoprecipitated with anti-IRS-1 antibody. TCL or IP were subjected to immunoblotting analysis with the indicated antibodies. (**c**) 3T3-L1 adipocytes were electroporated with mock vector and pFLAG-sDGKζ together with pGLUT4-myc-GFP, and GLUT4 translocation ratio was measured in these cells. The results are presented as the means ± S.E.M. of twenty cells. *p < 0.05 as compared with control group, ^#^*p* < 0.05 as compared with mock group. These are representative data from experiments independently performed at least three times.

To evaluate roles of the interaction between IRS-1 and DGKζ in GLUT4 translocation, the effect of insulin stimulation on the interaction was studied. Serum-starved CHO cells were stimulated with insulin for various times. Total cell lysates were immunoprecipitated by anti-IRS-1 antibody and immunoprecipitates were subjected to immunoblotting analysis with anti-DGKζ antibody. We detected the interaction of IRS-1 with DGKζ and PIP5K1α in the absence of insulin stimulation (Fig. 4b). These data suggested that PIP5K1α and DGKζ formed a ternary complex with IRS-1 without insulin stimulation. However in the insulin stimulated cell lysates, the interaction of IRS-1 with DGKζ or PIP5K1α was not observed, indicating that insulin stimulation dissociated this complex (Fig. 4a).

### Overexpression of sDGKζ (short-form DGKζ), which blocked the interaction of IRS-1 with DGKζ, enhanced GLUT4 translocation

As shown in Fig. 1c, the N-terminal region of DGKζ, which contained one C1 domain, was necessary and sufficient for the interaction with IRS-1. We prepared a construct expressing the GFP-tagged sDGKζ, which contained only this C1 domain and the effect of the sDGKζ overexpression on the interaction between DGKζ and IRS-1 was examined. FLAG-tagged full length DGKζ together with increasing amounts of GFP-sDGKζ were expressed in CHO cells, and the interaction between IRS-1 and FLAG-DGKζ was assessed. As shown in the left lane in Fig. 4b, we detected FLAG-DGKζ in the immunoprecipitates using the anti-IRS-1 antibody. However, when GFP-sDGKζ was over-expressed (Fig. 4b right lane), the interaction between IRS-1 and sDGKζ was observed but the interaction between IRS-1 and FLAG-DGKζ was inhibited. This indicated that GFP-sDGKζ overexpression competitively inhibited the interaction between IRS-1 and DGKζ. To evaluate the role of the interaction between IRS-1 and DGKζ in GLUT4 translocation, we overexpressed sDGKζ in 3T3-L1 adipocytes and GLUT4 translocation was measured in these cells. As shown in Fig. 4c, GLUT4 translocation was enhanced in sDGKζ overexpressed 3T3-L1 adipocytes both with and without insulin stimulation.

### DGKζ and PIP5K1α regulate each other’s activity

**Figure 5:**
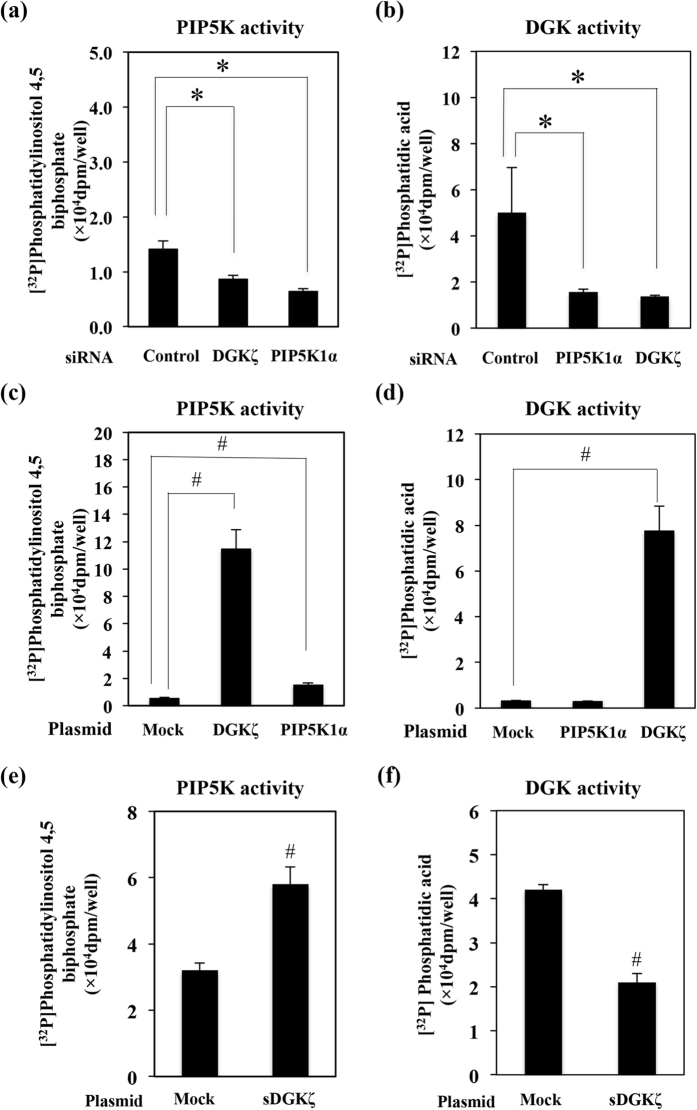
Effects of knockdown or overexpression of DGKζ or PIP5K1α on DGK or PIP5K activity. 3T3-L1 adipocytes were electroporated with non-relevant control siRNA (control), DGKζ siRNA, or PIP5K1α siRNA. Three days after electroporation, cell lysates were prepared to measure PIP5K activity (**a**) or DGK activity (**b**), respectively. Also, the CHO cells were transfected with mock vector or pFLAG-DGKζ plasmid, or pHA-PIP5K1α plasmid. Twenty four hours later, cell lysates were prepared to measure PIP5K activity (**c**) or DGK activity (**d**). CHO cells were transfected with mock vector or pGFP-sDGKζ plasmid for measuring the PIP5K and DGK activities (**e**,**f**). The methods to measure PIP5K or DGK activity were described in “Materials and Methods”. The results are presented at the means ± S.E.M. of three samples. These are representative data from experiments independently performed at least three times. The results are presented at the means ± S.E.M. of five samples. *p < 0.05 as compared with control group, ^#^*p* < 0.05 as compared with mock group.

We measured DGK or PIP5K activity in DGKζ or PIP5K1α knockdown cells. Fully differentiated 3T3-L1 adipocytes were electroporated with DGKζ or PIP5K1α siRNA and the electroporated cells were harvested to measure DGK or PIP5K activity. As shown in Fig. 5a, PIP5K activity was significantly suppressed in both DGKζ knockdown and PIP5K1α knockdown cells. In addition, DGK activity was also suppressed in both knockdown cells (Fig. 5b). Next, we overexpressed DGKζ or PIP5K1α in CHO cells and DGK or PIP5K activity in these cell lysates was measured. As shown in Fig. 5c, PIP5K activity was enhanced in PIP5K1α overexpressed CHO cells. Surprisingly, DGKζ overexpression enhanced PIP5K activity more strongly than PIP5K1α overexpression (Fig. 5c). On the other hand, DGKζ overexpression enhanced DGK activity in CHO cells, but PIP5K1α overexpression could not enhance DGK activity (Fig. 5d). Finally we measured DGK activity or PIP5K activity in the sDGKζ-overexpressing CHO cells. sDGKζ overexpression suppressed DGK activity whereas it significantly enhanced PIP5K activity (Fig. 5e,f).

## Discussion

IRSs are major substrates of insulin receptor tyrosine kinases. Phosphorylated tyrosine residues of IRSs bind to specific signaling molecules including the p85 PI 3-kinase regulatory subunit, resulting in activation of the downstream signals. Thus the interaction of IRSs with signaling molecules through recognition of tyrosine phosphorylation is believed to play critical roles in activation of the downstream signaling pathways. However, recently, we have reported that IRSs interact with various proteins (IRS-associated proteins; IRSAPs) even without insulin stimulation and form a high-molecular-mass complex (IRSome)^32^. We have already succeeded in identifying some IRSAPs, and we and others showed that IRSAPs have roles in modulation of insulin-induced IRS tyrosine phosphorylation (Nexillin, PHIP, 53BP2S, HSP90, Nedd4 and GKAP42)^10^,^11^,^12^,^13^,^14^,^15^, regulation of IRS-1 localization (AP-1)^29^ and regulation of RNA metabolism (PABPC1)^33^. In addition to these IRSAPs, in this paper, we identified DGKζ as a novel IRSAP by using yeast two-hybrid screening, and examined roles of DGKζ in GLUT4 translocation. DGKζ belongs to type IV of the DGK family of proteins and was reported to interact and form complexes with some enzymes, which are activated by DAG (Ras-GRP, cPKC) or PA (PIP5K1α)^25^,^26^,^27^. The function of PIP5K1α, one of the DGKζ binding proteins activated by PA in GLUT4 translocation, was also investigated.

The knockdown of DGKζ or PIP5K1α reduced insulin-induced GLUT4 translocation to the PM. Overexpression of DGKζ or PIP5K1α enhanced it. These results indicated that both DGKζ and PIP5K1α positively regulate the insulin-induced GLUT4 translocation. In addition, DGKζ knockdown, DGKζ overexpression or PIP5K1α overexpression did not affect the insulin canonical signals of the PI 3-kinase pathway (Supplemental Fig. S2), which are required for GLUT4 translocation, suggesting the existence of a novel pathway to regulate GLUT4 translocation. However, in PIP5K1α knockdown cells, Akt activation was obviously suppressed (Supplemental Fig. S2). This suppression might be caused by a shortage of a PI 3-kinase substrate, PI 4,5 P2 (PIP2), the product of PIP5K1α. Suppression of GLUT4 translocation in PIP5K1α knockdown cells might be also explained by the suppression of Akt phosphorylation. In contrast, PIP5K1α overexpression enhanced GLUT4 translocation in the absence of insulin. These data suggested that PIP5K1α has a function to enhance GLUT4 translocation in a PI 3-kinase independent pathway. Thus we concluded that DGKζ and PIP5K1α positively regulate GLUT4 translocation possibly through non-canonical insulin signaling.

**Figure 6:**
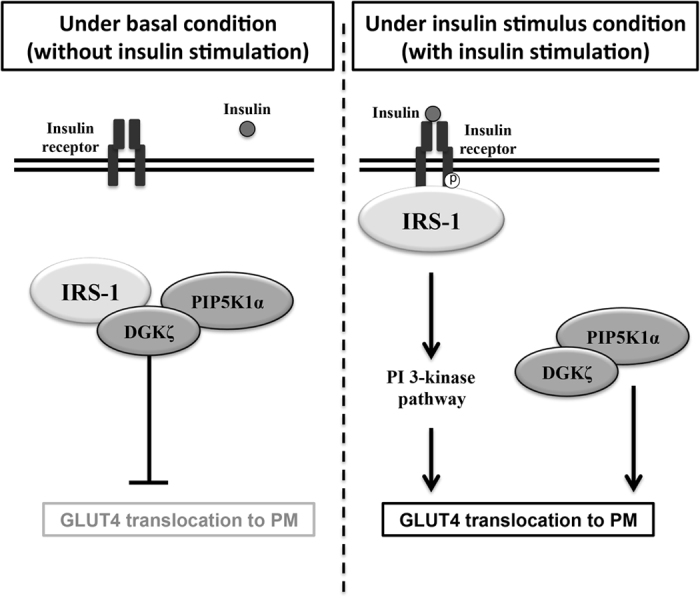
Working hypothesis of a novel regulatory mechanism of GLUT4 translocation by IRS-1-DGKζ-PIP5K1α complex. Our working hypothesis of regulatory mechanism of GLUT4 translocation to the PM is shown. In the absence of insulin (without insulin stimulation), insulin signal is not activated, and IRS-1 forms a ternary complex with DGKζ and PIP5K1α. The IRS-1-associated DGKζ inhibited GLUT4 translocation to the PM. In the presence of insulin, IRS-1 is tyrosine-phosphorylated by activated insulin receptor tyrosine kinases, and the DGKζ-PIP5K1α complex is dissociated from IRS-1. Then, IRS-1 can activate the canonical insulin signal, the PI 3-kinase pathway. In addition, the released DGKζ-PIP5K1α complex enhances GLUT4 translocation independently of PI 3-kinase pathway activation. In this paper, we show that both pathways are required for the GLUT4 translocation to the PM.

What is the role of the interaction between IRS-1 and DGKζ? Both DGKζ and PIP5K1α could be detected in the immunoprecipitates with anti-IRS-1 antibody, suggesting that IRS-1, DGKζ and PIP5K1α formed a ternary complex under basal conditions (without insulin stimulation). However, when the insulin signaling pathway was activated, the signals of DGKζ and PIP5K1α on the immunoblots disappeared, suggesting that DGKζ and PIP5K1α were released from IRS-1 by insulin stimulation (Fig. 4a). Overexpression of sDGKζ, which can competitively dissociate DGKζ from IRS-1 (Fig. 4b), enhanced GLUT4 translocation in 3T3-L1 adipocytes even in the absence of insulin (Fig. 4c), indicating that IRS-1-associated DGKζ inhibited GLUT4 translocation. The data showing that DGKζ knockdown enhanced GLUT4 translocation under basal conditions also supported the concept that IRS-1-associated DGKζ inhibited GLUT4 translocation without insulin stimulation (Fig. 6). It is possible that IRS-1-associated DGKζ inhibited GLUT4 translocation through DGK activity. On the contrary, PIP5K1α knockdown did not enhance GLUT4 translocation under basal conditions, suggesting that IRS-1-associated PIP5K1α did not function to inhibit GLUT4 translocation.

In DGKζ knockdown cells, under basal conditions (without insulin stimulation), GLUT4 translocation was enhanced but glucose uptake was suppressed (Fig. 2a,c). In addition, the fold stimulation of glucose uptake by insulin in DGKζ knockdown cells (2.258 ± 0.198) was almost comparable to that in control cells (2.056 ± 0.145). This shows uncoupling of GLUT4 translocation and glucose uptake. We have reported that chronic GH (growth hormone)^11^ pretreatment in 3T3-L1 adipocytes suppressed glucose uptake without affecting GLUT4 translocation to PM^34^. Other studies also have reported that a PI3K inhibitor (wortmannin) caused significant inhibition of insulin-stimulated glucose uptake, which did not prevent GLUT4 translocation in muscle cell and adipocytes^31^. From these data, others and we proposed the concept that GLUT4 translocation and fusion with PM are not sufficient to enhance glucose uptake *per se*, but that additional activation steps are required. This concept could explain an apparent uncoupling of GLUT4 translocation and glucose uptake in DGKζ knockdown cells. We also will in future experiments measure the glucose uptake in sDGKζ-overexpressing cells to show the uncoupling of GLUT4 translocation and glucose uptake in those cells. We attempted to overexpress sDGKζ by infection with sDGKζ-expressing lentivirus. However, due to the low level expression of exogenous sDGKζ, we could not detect the enhancement of glucose uptake in sDGKζ-expressing 3T3-L1 adipocytes. Much higher expression of sDGKζ might be required to enhance the glucose uptake. Further analysis is required to demonstrate the enhancement of glucose uptake by inhibiting IRS-1 and DGKζ interaction.

What is the role of the DGKζ or PIP5K1α dissociated from IRS-1? Since sDGKζ overexpression or insulin stimulation under which conditions DGKζ and PIP5K1α complex were dissociated from IRS-1, enhanced GLUT4 translocation, the DGKζ and PIP5K1α released from IRS-1 might function to enhance GLUT4 translocation to PM (Fig. 6). Overexpression of DGKζ, PIP5K1α or sDGKζ enhanced insulin-stimulated GLUT4 translocation. In all these cells, PIP5K activity was significantly activated. On the contrary, DGK activity was enhanced both in DGKζ and PIP5K1α overexpressing cells but not in sDGKζ overexpressing cells (Fig. 5). These data suggested that PIP5K activity in cells contributes to enhancement of GLUT4 translocation. We hypothesized that the dissociated DGKζ activates PIP5K1α, and PIP5K1α activity plays important roles in insulin-induced GLUT4 translocation.

In the present study, knockdown or overexpression of DGKζ decreased or enhanced PIP5K (Fig. 5a,c) and DGK activity (Fig. 5b,d), respectively. In addition, overexpression of PIP5K1α enhanced the PIP5K activity (Fig. 5c), but not the DGK activity (Fig. 5d). These results suggested that DGKζ may affect the PIP5K activity by PA generation, but PIP5K1α may not affect the DGKζ activity by PIP2 generation, consistent with a previous study^27^. In this study, we have shown that overexpression of sDGKζ inhibited the DGK activity but enhanced PIP5K activity (Fig. 5e). Because sDGKζ has a C1 domain, it is possible that sDGKζ competes with DGKζ for DAG. And sDGKζ enhances the PIP5K activity through an unknown mechanism that needs further investigation. Taken together, these findings raise the possibility of a direct linkage among DGKζ and PIP5K1α activation, and GLUT4 translocation via a PI-3 kinase-independent pathway.

How do DGKζ and PIP5K1α regulate GLUT4 translocation to PM independent of canonical insulin signaling? Many reports support the concept of an insulin stimulated cytoskeleton rearrangement via an actin-dependent pathway, not PI 3-kinase dependent pathway and this rearrangement could play an important role in GLUT4 translocation to the PM^35^,^36^. In addition, increased PIP2 due to overexpression of PIP5K1α promotes actin polymerization on the membrane-bound vesicles to and from motile actin comets, which inhibit the endocytosis of GLUT4^37^. Moreover, PIP2 regulated the GLUT4 exocytosis/endocytosis ratio via coating with filamentous-actin, neural Wiskott–Aldrich syndrome protein (N-WASP), dynamin, cortactin and caveolin on the GLUT4-containing vesicles. These findings suggested that PIP2 has a marked effect on the GLUT4 endocytosis, and intracellular vesicle traffic due to the change in actin dynamics^38^,^39^,^40^.

Recently there were some reports showing that diacylglycerol kinase proteins or activity are involved in the regulation of insulin bioactivity in different tissues. For example, knockdown of type I DGK (α, β, γ) proteins in pancreatic β-cells from male mice results in impairment of insulin secretion^41^. The reduced DGKδ protein expression is accompanied by the increased DAG level and elevated PKC activity, resulting in the impairment of IRS-1 tyrosine phosphorylation in skeletal muscle^42^,^43^,^44^. On the other hand, in the present study, we have shown that DGKζ regulates the GLUT4 translocation in 3T3-L1 adipocytes. Actually only DGKζ could interact with IRS-1 (Fig. 1d). In addition, DGKζ knockdown in 3T3-L1 adipocytes almost completely suppressed the DGK activity (Fig. 5b). Therefore, the dynamic regulatory mechanism of GLUT4 translocation through the IRS-1-DGKζ-PIP5K1α complex in 3T3-L1 adipocytes might be the unique role of DGKζ. It is possible that another DGK isoform has a special function to regulate the insulin signals and bioactivities in different tissues.

In summary, we found that DGKζ and PIP5K1α interact with IRS-1 without insulin stimulation. Under basal conditions, IRS-1-associated DGKζ inhibited GLUT4 translocation to the PM. Insulin stimulation dissociated the DGKζ and PIP5K1α from IRS-1, leading to enhancement of insulin-induced GLUT4 translocation through PIP5K activity (Fig. 6).

## Methods

### Materials

Dulbecco’s modified Eagle’s medium (DMEM), phosphate-buffered saline (PBS), and Hanks’ buffered salt solution were purchased from Nissui Pharmaceutical CO., (Tokyo, Japan). Calf serum (CS), fetal bovine serum (FBS) and bovine insulin were obtained from Sigma Aldrich (St. Louis, MO, USA). Penicillin and streptomycin were obtained from Banyu Pharmaceutical CO., (Ibaraki, Japan). Polyclonal anti-IRS-1 antibody was raised in rabbits as described previously^45^. All animal care and experiments conformed to the Guidelines for Animal Experiments of The University of Tokyo, and were approved by the Animal Research Committee of The University of Tokyo. Polyclonal anti-DGKζ antibody and anti-PIP5K1α antibody were kindly provided by Dr. MK Topham (University of Utah, Salt Lake, USA)^27^. Anti-IRβ antibody and anti-β-actin antibody were obtained from Santa Cruz Biotechnology, Inc. (Santa Cruz, CA USA). Anti-PI 3-kinase p85 subunit antibody, anti-Myc antibody (9E10) and anti-phosphotyrosine antibody (clone 4G10) were obtained from Millipore (Billerica, MA, USA). Anti-phospho-Akt (Ser-473) antibody and anti-Akt antibody were obtained from Cell Signaling Technology, Inc. (Danvers, MA, USA). Anti-FLAG antibody and anti-FLAG antibody-conjugated agarose beads were obtained from Sigma Aldrich. Horseradish peroxidase (HRP)-conjugated secondary anti-rabbit and anti-mouse IgG antibody were obtained from GE Healthcare (Pittsburgh, PA, USA). Enhanced chemiluminescence (ECL) reagents were from PerkinElmer Life Science (Boston, MA, USA). Alexa Fluor 594-conjugated secondary anti-mouse IgG antibody was obtained from Invitrogen (Carlsbad, CA, USA). Protein A-Sepharose was purchased from PerkinElmer (Waltham, MA, USA). Control, DGKζ and PIP5KIα specific siRNAs were purchased from RNAi CO. (Tokyo, Japan). The sequence of the DGKζ siRNA used was 5′-CCA ACG UGU CCG GUG ACU UCU-3′. The sequence of the PIP5KIα siRNA used was 5′-CUU GCC UCG GUC AGU CAA AAU-3′. The nonrelevant control siRNA sequence was 5′-GUA CCG CAC GUC AUU CGU AUC-3′. Other chemicals were of the reagent grade available commercially.

### Plasmids

pAS-IRS-1 was prepared as described previously^11^ and used for two-hybrid screening as bait. The FLAG tagged full-length DGKζ, HA tagged PIP5K1α, GFP tagged DGKα, GFP tagged DGKγ, HA tagged DGKε, HA tagged DGKβ, GFP tagged DGKδ, HA tagged DGKι and deletion series of DGKζ expressing plasmids were a gift from Dr. MK Topham. The short-form DGKζ (sDGKζ), which contains C1 domain, and was constructed as follows. The C1 domain of DGKζ were generated by PCR using FLAG-DGKζ as a template. Amplified fragments were digested by EcoR1 and BglII and inserted into pCMV-FLAG or pEGFP-C1 vector in-frame. The exofacial myc-tagged GLUT4-EGFP was kindly provided by Dr. J. E. Pessin^34^.

### Yeast two-hybrid screening

Yeast two-hybrid screening was performed as described previously^11^. Yeast strain AH109 was purchased from TaKaRa Bio Company (Japan).

### Cell cultures

HEK293T cells and CHO cells were cultured as described previously^11^. Murine 3T3-L1 preadipocytes were purchased from the American Type Tissue Culture Collection. 3T3-L1 preadipocytes were cultured in DMEM containing 10% calf serum at 37 °C in 5% CO_2_ atmosphere and induced to differentiate into adipocytes as described^11^.

### Transient transfection of HEK293T cells, CHO cells or 3T3-L1 adipocytes

HEK293T cells or CHO cells were transiently transfected with expression plasmids by lipofectamine 2000 according to the manufacture protocol (Invitrogen, USA) or by the polyethyleneimine method as described before^29^. Transient transfection of 3T3-L1 adipocytes was described previously^15^. In some experiments, the electroporated adipocytes were seeded on coverslips.

### GLUT4 translocation assay

Differentiated 3T3-L1 adipocytes electroporated with siRNA or plasmid along with pGLUT4-myc-green fluorescent protein (GFP) were grown on coverslips. Twenty-four hours after electroporation, cells were serum-starved for 4 h and treated with or without insulin for 20 min. Cells were then fixed using 4% paraformaldehyde for 10 min without permeabilization, blocked with 3% bovine serum albumin for 1 h at room temperature. Coverslips were immunostained with anti-Myc antibody for 1 h at 37 °C. After incubation, the coverslips were washed three times with PBS solution, followed by incubation with Alexa Fluor 594-labeled goat anti-mouse IgG for 1 h at 37 °C. Coverslips were again washed three times with PBS and mounted in VECTASHIELD medium. PM localization was determined by using confocal fluorescence microscopy (OLYMPUS, Tokyo, Japan) to score 20 representative cells per condition for the appearance of a PM ring of GLUT4. The ratio of GLUT4 translocation was calculated by myc fluorescence on PM/GFP fluorescence in whole cells.

### Glucose uptake assay

Differentiated 3T3-L1 adipocytes were incubated with the indicated concentration of insulin in Krebs-Ringer phosphate (KRP) buffer (20 mM Hepes, 140 mM NaCl, 5 mM KCl, 2.5 mM MgSO_4_, 1 mM CaCl_2_, 1% BSA, pH7.4) for 20 min at 37 °C. Then 0.1 mM 2-deoxy-D-glucose containing 10 μCi/ml 2-deoxy-D-[2,6-^3^H] glucose was added, and cells were incubated for 4 min at 37 °C. The reaction was terminated by addition of ice-cold PBS containing 10 mM D-glucose. Cells were lysed with 0.1 N NaOH, and radioactivity taken up by cells was measured by a liquid scintillation counter.

### Immunoprecipitation followed by immunoblotting

Cells were lysed at 4 °C with ice-cold Tris/TritonX-100 lysis buffer [50 mM Tris-HCl pH 7.4, 150 mM NaCl, 1 mM EDTA, 1% TritonX-100, 20 μg/ml phenylmethylsulfonylfluoride (PMSF), 5 μg/ml pepstatin, 10 μg/m leupeptin, 100 KIU/ml aprotinin, 1 mM Na_3_VO_4_, and 10 mg/ml *p*-nitrophenylphosphate (PNPP)]. Insoluble materials were removed by centrifugation at 15,000 × *g* for 10 min at 4 °C and supernatant was prepared as total cell lysates. For immunoprecipitation, 1 mg protein of total cell lysates were incubated with the indicated antibody for 2 h at 4 °C and the immunocomplexes were precipitated with 10 μl of protein A-Sepharose for polyclonal antibody. These precipitates were washed 3 times with ice-cold lysis buffer. These precipitates or total cell lysates were subjected to SDS-polyacrylamide gel electrophoresis (SDS-PAGE) and immunoblotted with indicated antibody.

### DGK activity assay

DGK activity was measured in the whole cell lysates by octylglucoside mixed micelle assay^25^. The cells were lysed with lysis buffer (20 mM Tris-HCl pH7.4, 0.25 M sucrose, 1 mM DTT), and then the cells were sonicated for 5–10 sec. The cell lysates were immunoprecipitated with indicated antibody. Immunoprecipitates were washed once with lysis buffer, LiCl buffer (100 mM Tris-HCl, pH 7.4, and 500 mM LiCl), distilled water, and TNE buffer (10 mM Tris-HCl, pH 7.4, 150 mM NaCl, and 1 mM EDTA) and finally resuspended in 40 μl of reaction buffer (50 mM MOPS, pH7.2, 20 mM NaF, 1 mM DTT). A kinase reaction was initiated by incubation of the reaction mixture (50 μl total) containing 1 mM 1,2 diacyl-*sn*-glycerol-3-phospho-L-serine, 50 mM MOPS, 20 mM NaF, 1 mM DTT, 50 mM octylglucoside in the presences of 1 mM [γ-^32^P]ATP at 25 °C for 20 min. The reaction was stopped by adding 1 M HCl. The 10 μl PA solution (2.5 mg/mL) and 250 μl elution solution [CHCl_3_/MeOH (1:1)] were added into the reaction mixture. After the centrifugation, the lower layer of solution was taken for measuring DGK activity. A lipid product was spotted onto a silica gel plate, and developed with ethyl acetate/2.2.4.-trimethyl pentane/acetic acid/H_2_O (45:25:10:5). The DGK activity was visualized by autoradiography (FLA-5000 image system, Fuji Photo Film Co., Ltd.). Also, the images were quantified using the Image Reader and Image Gauge (Fuji Photo Film Co., Ltd.).

### PIP5K activity assay

PIP5K assays were performed in 50 μl reaction mixtures, containing 50 mM Tris, pH 7.5, 100 mM NaCl, 0.5 mM EGTA, 1 mg/mL PI(4)P (phosphatidylinositol-4-phosphate/CHCl_3_) and 50 μM [γ-^32^P]ATP (1 μCi/assay) (27). The reaction mixture was preincubated at 25 °C for 5 min, and the reactions were initiated by the addition of the 10 μl of cell lysate. After 20 min incubation, the reaction was stopped by adding 100 μl of 1 M HCl. The samples were centrifuged for 2 min, and then the lower layer of each sample was taken to examine the PIP5K activity. A lipid product was spotted onto a silica gel plate, and developed with ethyl acetate/2.2.4.-trimethyl pentane/acetic acid/H_2_O (45: 25:10:5). The PIP5K activity was visualized by autoradiography. Also, the images were quantified using the Image Reader and Image Gauge.

### Statistical analysis

Data are expressed as mean ± S.E.M. Comparisons between two groups were performed using Student’s t-test, whereas comparisons among more than two groups were analyzed by one-way or two-way ANOVA and the Tukey post hoc test. Values of *P* < 0.05 were considered statistically significant.

## Additional Information

**How to cite this article**: Liu, T. Y. *et al*. A novel IRS-1-associated protein, DGKζ regulates GLUT4 translocation in 3T3-L1 adipocytes. *Sci. Rep.* **6**, 35438; doi: 10.1038/srep35438 (2016).

## Supplementary Material

Supplementary Information
