# Supplementary material for: A novel IRS-1-associated protein, DGKζ regulates GLUT4 translocation in 3T3-L1 adipocytes
Source: Sci Rep. 2016 Oct 14;6:35438. doi: 10.1038/srep35438 (PMC5064357; doi:10.1038/srep35438)
Supplement: Supplementary Information [file srep35438-s1.pdf]

A novel IRS-1-associated protein, DGKζ regulates GLUT4 translocation in 3T3-L1 adipocytes

TingYu Liu[1], BuChin Yu[1], Mamoru Kakino[1], Hitoshi Fujimoto[1], Yasutoshi Ando[1], Fumihiko Hakuno[1]*, Shin-Ichiro Takahashi[1+]

[1]Departments of Animal Sciences and Applied Biological Chemistry, Graduate School of Agriculture and Life Sciences, The University of Tokyo, 1-1-1 Yayoi, Bunkyo-ku, Tokyo, Japan

* **Corresponding author:** Fumihiko Hakuno, Ph.D, Departments of Animal Sciences and Applied Biological Chemistry, Graduate School of Agriculture and Life Sciences, The University of Tokyo, 1-1-1 Yayoi, Bunkyo-ku, Tokyo, Japan

TEL: +81-3-5841-1310; FAX: +81-3-5841-1311; E-mail: ahakuno@mail.ecc.u-tokyo.ac.jp

[+] **Co-corresponding author:** Shin-Ichiro Takahashi, Ph.D, Departments of Animal Sciences and Applied Biological Chemistry, Graduate School of Agriculture and Life Sciences, The University of Tokyo, 1-1-1 Yayoi, Bunkyo-ku, Tokyo, Japan

TEL: +81-3-5841-1310; FAX: +81-3-5841-1311; E-mail: atkshin@mail.ecc.u-tokyo.ac.jp

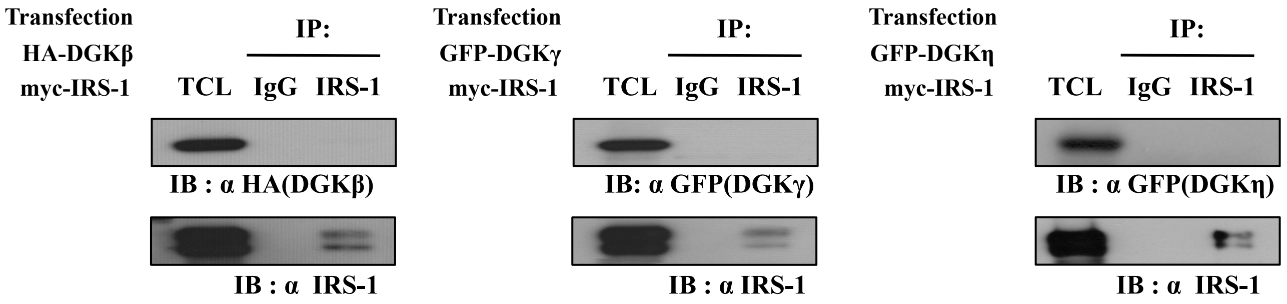

Supplemental Figure S1 The plasmid of HA-DGKβ, GFP-DGKγ, or GFP-DGKη was co-expressed with myc-IRS1 in HEK293T cells, respectively. Cells were serum-starved for 4 h and cell lysates were prepared (total cell lysate, TCL). Cell lysates were immunoprecipitated by anti-IRS-1 antibody. TCL and immunoprecipitates (IP) were subjected to immunoblotting analysis with indicated antibodies.

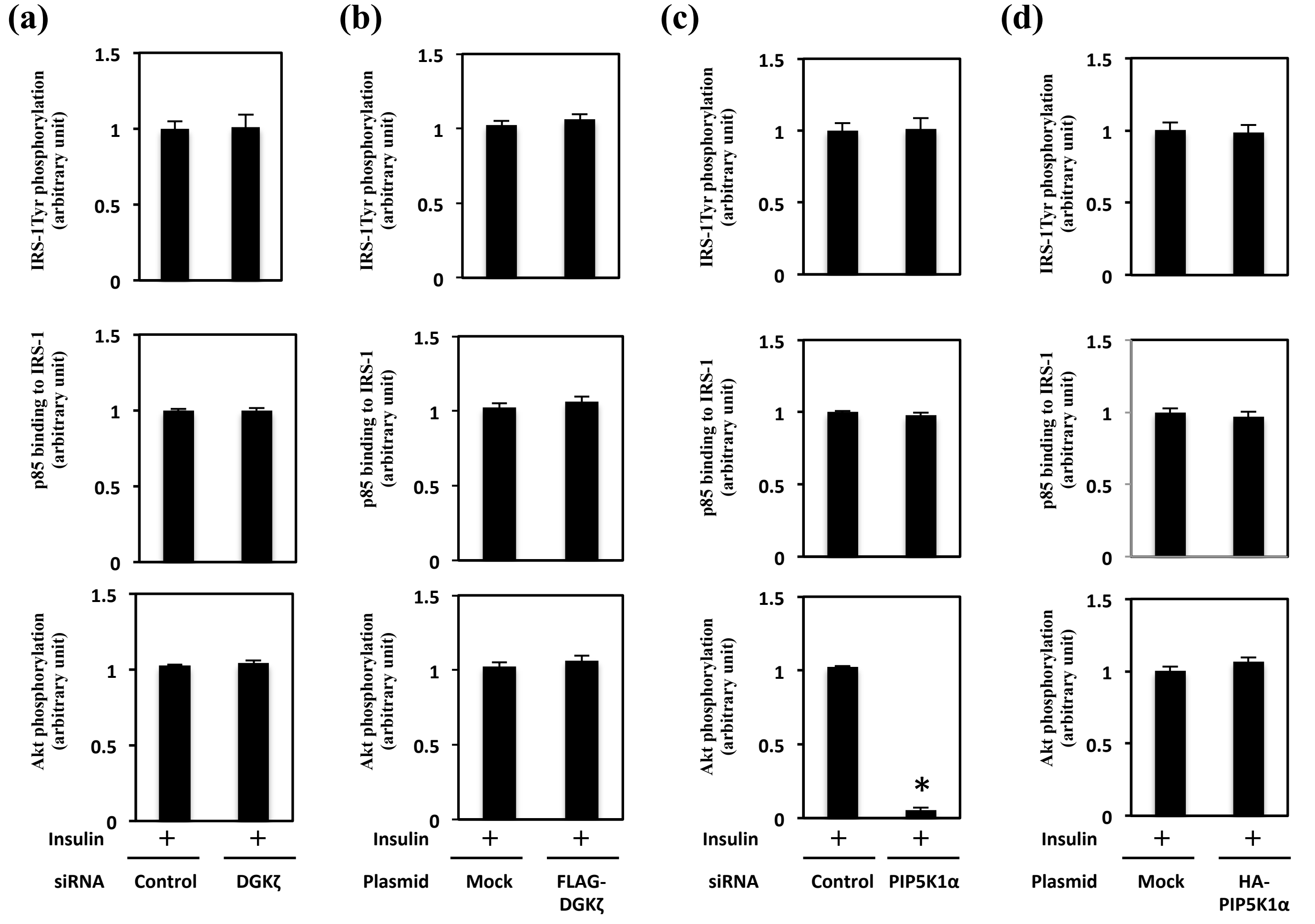

Supplemental Figure S2

(a, c) Fully differentiated 3T3-L1 adipocytes were electroporated with non-relevant control siRNA (control) or DGKζ siRNA or PIP5K1α siRNA. And insulin signal was examined as described in Figure 2b and 3b. Bands from Figure 2b and Figure 3b were quantified from each blot by NIH Image J software. The results are presented at the means ± S.E.M. of three different experiments. * $p < 0.05$ as compared with control group plus insulin treatment.

(b, d) CHO cells were transfected with mock vector, pFLAG-DGKζ or pHA-PIP5K1α plasmid, respectively. And insulin signal was examined as described in Figure 2e and 3e. Bands from Figure 2e and Figure 3e were quantified from each blot by NIH Image J software. The results are presented at the means ± S.E.M. of three different experiments.
